# Receptor Pre-Clustering and T cell Responses: Insights into Molecular Mechanisms

**DOI:** 10.3389/fimmu.2014.00132

**Published:** 2014-04-30

**Authors:** Mario Castro, Hisse M. van Santen, María Férez, Balbino Alarcón, Grant Lythe, Carmen Molina-París

**Affiliations:** ^1^Grupo de Dinámica No-Lineal and Grupo Interdisciplinar de Sistemas Complejos (GISC), Escuela Técnica Superior de Ingeniería (ICAI), Universidad Pontificia Comillas, Madrid, Spain; ^2^Departamento de Biología Celular e Inmunología, Centro de Biología Molecular Severo Ochoa, Consejo Superior de Investigaciones Científicas, Universidad Autónoma de Madrid, Madrid, Spain; ^3^Department of Applied Mathematics, School of Mathematics, University of Leeds, Leeds, UK

**Keywords:** T cell receptor, clustering, stochastic dynamics, signaling, naive T cells, memory T cells

## Abstract

T cell activation, initiated by T cell receptor (TCR) mediated recognition of pathogen-derived peptides presented by major histocompatibility complex class I or II molecules (pMHC), shows exquisite specificity and sensitivity, even though the TCR–pMHC binding interaction is of low affinity. Recent experimental work suggests that TCR pre-clustering may be a mechanism via which T cells can achieve such high sensitivity. The unresolved stoichiometry of the TCR makes TCR–pMHC binding and TCR triggering, an open question. We formulate a mathematical model to characterize the pre-clustering of T cell receptors (TCRs) on the surface of T cells, motivated by the experimentally observed distribution of TCR clusters on the surface of naive and memory T cells. We extend a recently introduced stochastic criterion to compute the timescales of T cell responses, assuming that ligand-induced cross-linked TCR is the minimum signaling unit. We derive an approximate formula for the mean time to signal initiation. Our results show that pre-clustering reduces the mean activation time. However, additional mechanisms favoring the existence of clusters are required to explain the difference between naive and memory T cell responses. We discuss the biological implications of our results, and both the compatibility and complementarity of our approach with other existing mathematical models.

## 1 Introduction

A hallmark of the adaptive immune system is the ability of T cells, making use of the T cell receptors (TCRs) on their surface, to recognize a given agonist peptide–MHC ligand complex (pMHC) with high sensitivity (1). Some aspects of TCR–pMHC molecular interactions that are of current research interest are the frequency of encounters between T cells and the agonist pMHC, how cell–cell interactions determine the activation of lymphocytes (2), how early interactions change the state of the T cell receptor (3), what are the mechanisms of modulation of receptor–ligand interactions at cell–cell interfaces (4), and how protein organization in the cell membrane (for instance, protein islands or lipid rafts) affects the recognition process (5). Some recent experiments have explored the role of dimensionality on T cell activation and have highlighted the significance of the events taking place at the receptor level [see Refs. (1) and (6) for comprehensive reviews].

These open questions have been addressed with the use of mathematical modeling. Different theories can be classified according to the level of description (7). At the individual TCR–pMHC bond level, the kinetic proof-reading model (8) assumes that the TCR needs to undergo a series of consecutive (phosphorylation) steps before being triggered. Also at the TCR level, the optimal dwell time model (9) reconciles the concurrence of different timescales, providing an *optimal* timescale between the very short times related to the off rate of TCR–pMHC binding, and the long times related to kinetic proof-reading mechanisms. The TCR occupancy model (10) considers the cell as a *counting device* in which multiple TCR–pMHC interactions are required to activate a T cell. In a similar fashion, the serial triggering model (11) proposed that the same pMHC can engage *serially* different TCRs. This model enriches the viewpoint of the TCR occupancy model, by giving greater relevance to the role of the pMHC itself. Finally, the serial encounter model (12) and the confinement time model (13) combine several of the ideas above and provide some appealing explanations by relaxing some restrictions in those models.

While antigen presenting cells (APCs), such as dendritic cells or B cells, present 10^3^–10^4^ times more self-pMHC than antigenic pMHC, self-pMHC ligands by themselves do not usually elicit a T cell response, even though their affinity for TCR*αβ* is only 10 times lower than the affinity of the antigenic pMHC (14). This illustrates how a small difference in affinity results in high specificity, when there is only a few antigenic pMHC molecules in a background of self-pMHC ligands (15).

The T cell signaling process begins with (extracellular) TCR–pMHC binding, followed by phosphorylation of the intracellular ITAM domains of the TCR–CD3 complex. When a TCR binds a pMHC molecule, the TCR*αβ* hetero-dimer binds the peptide, while the CD4 or CD8 co-receptor binds the MHC molecule. The binding of the co-receptor activates the tyrosine kinase Lck, which phosphorylates the ITAMs of the CD3 complex. ITAM phosphorylation allows recruitment of intracellular signaling components that mediate downstream signaling events (16).

It has recently been suggested that, contrary to what happens in TCR micro-clusters and the immunological synapse, clustering is not only induced by the ligand but by an *avidity maturation* mechanism (or pre-clustering) (17), allowing the aggregation of chains of TCRs as long as 20 units (around 200 nm long), and referred to as *nano-clusters* (3, 18). Specifically, multimeric TCR–CD3 complexes are activated at low agonistic pMHC concentrations and monomeric TCRs remain unaffected at low ligand concentration. The TCR nano-clusters could enhance T cell sensitivity by the mechanisms proposed in the models of T cell activation (7), as their existence would reduce the time needed for two (or more) receptors to aggregate (by diffusion). This pre-cluster formation could be explained by three different mechanisms (3):

- Multimeric complexes (or clusters) enhance the TCR *avidity* toward the ligand, which is expressed in clusters on the surface of APCs (19–21). At low ligand concentration, only multimeric TCR clusters are bound to ligand, as TCR monomers require higher ligand concentration. Monomeric TCRs might only be activated at high agonist doses.
- Multimeric complexes allow the propagation of the activation signal from ligand-bound TCR*αβ* to neighboring receptors in the same TCR *multimer*.
- Linear arrays of multimeric TCR complexes help a single pMHC serially trigger several receptors (11).

The existence of these nano-clusters does not exclude additional mechanisms of T cell activation, as long as they involve the *cooperation* of receptors when they aggregate. Thus, while models such as kinetic proof-reading [and improvements as described in Ref. (22)] operate at the level of a single receptor, other models might be used in combination with the fact that the pre-cluster distribution of naive and memory T cells is different.

Additionally, the fact that the TCR stoichiometry has not been resolved under physiological conditions, yet, makes it even more difficult to understand, at a molecular level, the dynamics of TCR pre-clustering (23). TCR pre-clustering could be an example of a more general mechanism of membrane-bound molecular pre-clustering, as clustering prior to cell–cell interaction has also been observed on the surface of APCs (19–21). It is worth mentioning that monomeric TCRs can still be activated at increasing ligand concentrations, thus, conferring the T cell with a capacity to generate a dose-dependent response at very high pMHC doses, when multimeric TCR–CD3 complexes are already saturated (18). Such mechanisms have been previously described for chemotactic bacteria, as a cellular mechanism to control sensitivity (24).

**Figure 1:**
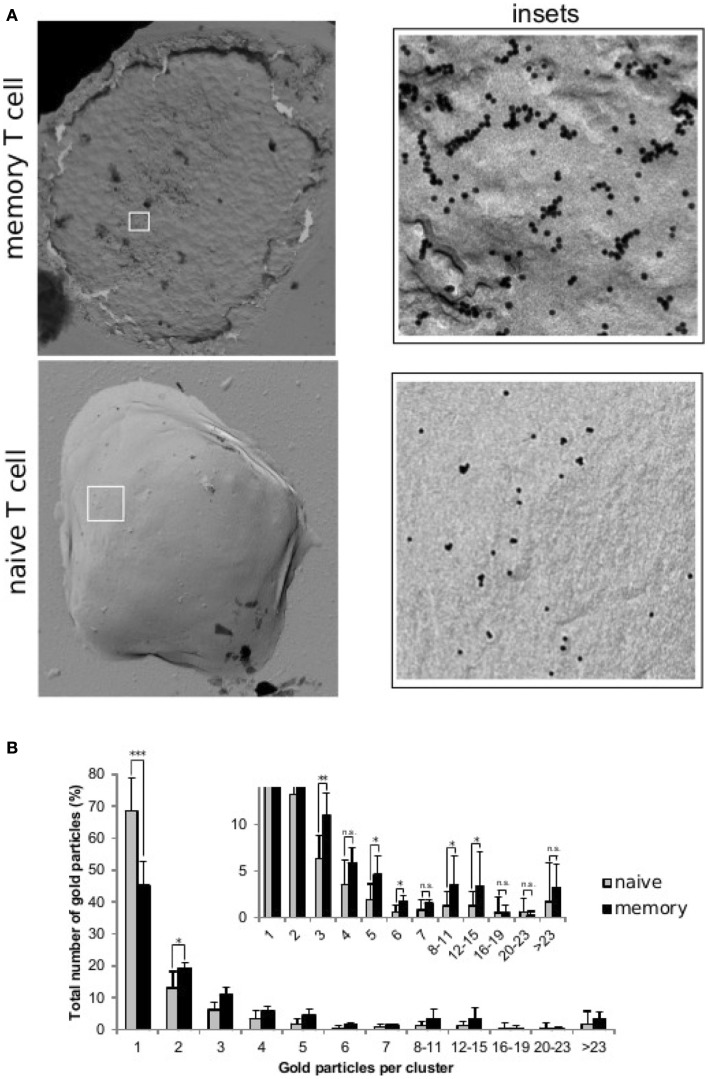
**Distribution of TCRs at the surface of naive and memory T cells**. Resting naive and memory CD8^+^ OT-1 T cells were labeled with the CD3ϵ-specific mAb 2C11 and 10 nm gold-conjugated protein-A. Cell surface replicas of the labeled T cells were analyzed by transmission electron microscopy and the number and size of the observed gold clusters were recorded. **(A)** TEM image of surface replicas of a memory and a naive OT-1 T cell. The insets to the right show an enlargement of the boxed areas. **(B)** Quantification (mean ± SD) of gold particles in clusters of the indicated sizes for resting naive T cells (gray bars, 7 cells, 9190 particles) and memory T cells (black bars, 5 cells, 3001 particles). The inset shows a detailed view of the distribution of clusters of three or more gold particles and statistical analysis (2-tailed Student’s *t*-test: *p < 0.05, **p < 0.01, and ***p < 0.001). All naive and memory T cells had clusters with gold. However, whereas in naive T cells the maximum gold cluster size shared by all cells was four, this was eight for memory T cells. Also clusters bigger than twenty three particles were present in four out of five memory T cells, and only two out of seven naive T cells.

Various mechanisms have already been suggested, at the population, cellular or molecular level, to explain the capacity of T cells to respond, faster and more strongly, to a second antigenic encounter. However, the underlying mechanisms of the observed changes in the sensitivity of the T cell for pMHC ligand-mediated TCR stimulation (25) have not yet been clearly elucidated. Interestingly, the distribution of clusters in naive and memory T cells is different: memory T cells accommodate larger linear TCR clusters than naive ones. This could explain why memory T cells elicit more rapid responses than naive T cells (17) (see Figure 1 below).

In this paper, we explore the consequences of TCR pre-clustering in signaling and in distinguishing naive from memory T cell responses. We present some experimentally obtained distributions of TCR clusters for both types of cells (see Figure 1), and two complementary theoretical models: (i) a simple model of receptor oligomerization that describes cluster size distributions, and (ii) a generalization of the stochastic T cell response criterion of Ref. (26), to accommodate the hypothesis that the minimum signaling unit is composed of a TCR receptor cluster that is bound by the same cross-linked multivalent ligand. We find that this signaling unit is able to discriminate between agonist and antagonist pMHC ligands (with greater sensitivity than in the monomeric case), and to explain some of the advantages that higher cluster sizes can provide to memory T cells. The model also points at the need to invoke additional cooperativity mechanisms, to explain the experimentally observed role of clustering in T cell responses (27). Finally, this model of ligand-induced TCR cross-linking can be relevant in physiological conditions, according to the defective ribosomal products (DRiP) hypothesis (28, 29), which provides a rapid source of peptide precursors to optimize immuno-surveillance of pathogens and tumors (30).

## 2 Mathematical Modeling of TCR Pre-Clustering and T Cell Activation

### 2.1 Model 1: T cell receptor pre-clustering

The TCR–CD3 complex consists of the pMHC binding TCR*αβ* hetero-dimer, associated with the hetero-dimers CD3*γϵ* and CD3*δϵ*, and the homo-dimer CD3*ζζ*. Binding of a stimulating pMHC ligand by the extracellular domain of TCR*αβ* results in conformational changes in the intracellular part of the CD3*ϵ* chain, and phosphorylation of the immuno-receptor tyrosine-based activation motifs (ITAMs) in the intracellular domains of the CD3*γϵ*, CD3*δϵ*, and CD3*ζζ* dimers, which in turn lead to initiation of downstream signaling cascades and T cell activation.

It has long been recognized that the TCR–CD3 complex forms clusters upon ligand binding (31–36). More recently, it has been shown that in the absence of stimulating pMHC ligand, TCR–CD3 complexes are already expressed at the cell surface as a combination of monomeric and oligomeric TCR complexes or TCR nano-clusters (18). Electron microscopy (EM) analysis of immuno-gold-labeled human and murine T cells showed that these nano-clusters consist of up to 20 TCR–CD3 complexes. The exact stoichiometry of the nano-clusters has not been resolved yet.

The integrity of TCR nano-clusters depends on cholesterol present at the cell surface membrane (18). The formation of the clusters depends, at least, on the trans-membrane region of the CD3*ζζ* homo-dimer (17), perhaps due to the capacity of *ζζ* dimers to form dimers of dimers (37). Other possible mechanisms of cluster formation rely on the capacity of the extracellular domain of TCR*α* to dimerize (38).

This body of experimental evidence allows us to conclude that multimeric TCR–CD3 complexes are co-expressed with TCR monomers on the surface of resting T cells.

**Figure 2:**
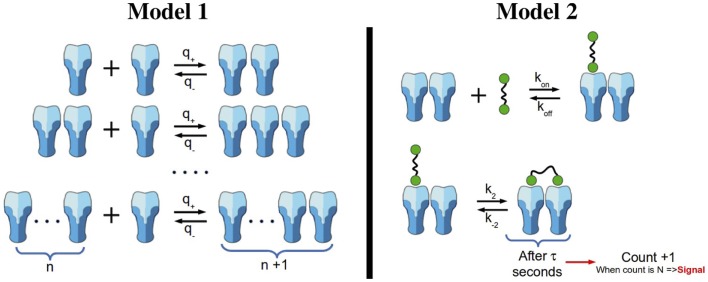
**Oligomerization and signaling models**. Left panel: oligomerization model not mediated by ligand (Model 1). We assume that receptors are able to diffuse and aggregate to an existing cluster. However, we exclude the possibility of clusters with size larger than one to diffuse. Clusters grow one monomeric unit at a time. Right panel: reactions included in the stochastic activation model (Model 2). Ligands in solution are able to attach monovalently to any receptor in a cluster (top reaction). In addition, ligand-induced TCR cross-linking can occur once a ligand is bound to a TCR in a given nano-cluster (bottom reaction). Following Ref. (26), once the bivalently bound ligand has been attached for a time *τ*, we count that state as a signaling unit. After *N* of these units have been generated, the cell becomes activated.

A simple model of aggregation of TCR*αβ* units is depicted in the left panel of Figure 2. Given a chain of length *n* (with *n* hetero-dimers linked), in a small time interval Δ*t*, with probability *q*_+_ Δ*t*, the chain increases to length *n *+ 1, and with probability *q*_−_ Δ*t*, the chain decreases to length *n *− 1. Thus, by probability conservation, the probability to remain the same length *n* is 1 − (*q*_+_ + *q_−_*)Δ*t*.

Mathematically, the dynamics of the process can be described by a continuous time Markov chain (39) (or birth and death process, as we assume that polymerization takes place in unit steps). The state space is {1, 2, 3, …, *n* − 1, *n*, *n* + 1, …}, where the number denotes the number of TCRs in a cluster:

$$1\overset{q_{-}}{\underset{q_{+}}{⇋}}2\overset{q_{-}}{\underset{q_{+}}{⇋}}3\overset{q_{-}}{\underset{q_{+}}{⇋}}\cdots \overset{q_{-}}{\underset{q_{+}}{⇋}}n-1\overset{q_{-}}{\underset{q_{+}}{⇋}}n\overset{q_{-}}{\underset{q_{+}}{⇋}}n+1\cdots \;.$$

The forward Kolmogorov equations for the probability of having a cluster of size *n* are given by (40)

$$\begin{array}{llll} \frac{dp_{n}\left(t\right)}{dt} & =q_{+}p_{n-1}\left(t\right)+q_{-}p_{n+1}\left(t\right)-\left(q_{+}+q_{-}\right)p_{n}\left(t\right),\forall n\geq 2,\; & & \;\\ \frac{dp_{1}\left(t\right)}{dt} & =q_{-}p_{2\left(t\right)}-q_{+}p_{1}\left(t\right).\; & & \; \end{array}$$

The stationary probability distribution is then given by

(1) $$\begin{array}{llll} & \underset{t\to +\infty }{\lim}\;p_{n}\left(t\right)\equiv \pi_{n}=\frac{b^{n-1}\left(1-b\right)}{\left(1-b^{N_{\max}}\right)}\;,\; & & \;\\ & \;b<1\;,\;n\in \left\{1,2,3,\ldots ,N_{\max}\right\}\;, \end{array}$$

with $b=\frac{q_{+}}{q_{-}}$, and *π_n_* the probability (in thermodynamic equilibrium) to have a cluster of size *n*. When *b* < 1 (the number of clusters with a given size, *n*, decreases as *n* increases), and taking into account that peripheral T cells have around $N_{\max}≃3\times 10^{4}$ receptors, the latter expression can be further simplified to

(2) $$\pi_{n}=b^{n-1}\left(1-b\right)\;,\;b<1\;,\;n\in \left\{1,2,3,\ldots \right\}\;.$$

### 2.2 Model 2: A bivalent model for T cell activation

The TCR–pMHC binding model introduced in Ref. (26) considered monovalent pMHC ligands binding to TCR monomers on the surface of a T cell. Monovalent ligands have been reported to elicit a T cell response (41–43), but only when they are immobilized on a surface (which makes it difficult to assess whether they are truly monovalent or not). Yet, multivalent receptor–ligand interactions are required to elicit T cell responses in both CD4^+^ and CD8^+^ T cells. In what follows, and supported by a body of experimental work (3, 24, 44), we adopt the hypothesis that the minimum activating unit is a TCR–pMHC cross-linked dimeric complex (31, 45–47). We make use of the binding model (Model 2) with pMHC dimers (ligands) and dimeric TCRs (receptors), described in the right panel of Figure 2.

Gold-labeling experiments support the existence of nano-clusters with more than two TCRs, yet it can be shown (see Section 5.2) that the key parameter of the mathematical model is the fraction of monomeric to multimeric TCR clusters. Thus, without loss of generality, we will assume that all TCR clusters are dimeric.

The biochemical reactions encoded by the right panel of Figure 2 are as follows:

- A (bivalent) ligand can bind a free receptor with monomeric binding reaction rates (*k*_on_ and *k*_off_). Although not shown in the figure, we allow for a second ligand to bind the free receptor of the cluster. However, at low concentrations of ligands, this reaction can be safely neglected.
- Cross-linking of a singly bound ligand follows with rates *k*_2_ (forward reaction) and *k*_−2_ (backward reaction).
- If the complex formed by the ligand cross-linked to the dimeric TCR cluster lasts at least a time *τ*, *dwell time*, we count that event. When we reach *N* such events, we will assume that a T cell response is initiated. The rationale behind this T cell response criterion follows the work of Palmer et al. (48), where the concepts of minimum dwell time and productive binding were introduced. This model combines aspects of the kinetic proof-reading (8) and the serial triggering models (7, 11). The minimum dwell time for a TCR–pMHC complex is the time the complex must remain bound in order to reach a level of ITAM phosphorylation, which will allow TCR triggering. Any binding, which persists for longer than the minimum dwell time is classified as a productive binding [see Refs. (48) and (26) for further details].
- From an immunological perspective, the relevant parameter is the *mean time to signal initiation*, or MTSI (26). Namely, the MTSI is the average time needed for a T cell response according to the criterion that at least *N* TCR dimers should be bivalently engaged to a bivalent ligand (pMHC) for at least a time *τ*.

Here we assume that *N* is around 10–100. That is, 10–100 TCRs are required for signaling and *N_B_* = *b *× *N_R_* is of the order of 10^4^, with *N_B_* the total number of clusters on the T cell surface. This means, under the assumptions of Model 2, that at most, there can be *N* = 100 internalization events, as this is the number of triggered TCRs. Thus, in this approximation, the loss of TCR due to internalization after triggering can be safely neglected. Nevertheless, internalization is an important step in early signaling, and a proper mechanistic model to justify the value of *τ* will require internalization to be considered. This analysis is out of the scope of this article.

We implement these reactions as a Markov process, and solve them numerically using the standard Gillespie algorithm (49), and with the parameters summarized in Table 1. We have made use of three different ligands: 4A, 4P, and 4N, which were also used in Ref. (26). For these ligands, that bind the same TCR with different affinities, a simple estimation of the number of cross-linking events required to elicit a T cell response is summarized in Table 2.

**Table 1:** **Summary of the parameters used in the stochastic simulations**.

Parameter	Value	Comment
*N_A_*	6.023 × 10^23^	Avogadro’s number
*N_R_*	30,000	Average number of TCRs per T cell (34 )
*V*	50 μl	Volume of the experiment
*N_C_*	10^5^ cells	Number of T cells in the experiment
*V_C_*	*V*/*N_C_*	Average extracellular volume per cell
*k*_−2_	*k*_off_	Cross-linking off rate
*k*_2_	$k_{\mathrm{off}}\left(k_{d}∕k_{d}^{\mathrm{dimer}}\right)$	Cross-linking rate^a^
*N*	10	Minimum number of bound dimer-bivalent clusters to elicit a T cell response
τ	1–4 s	Dwell time

*For typical values of the dissociation rate, *k_d_*, we find that *k*_2_ is about 10–50 times *k*_off_. We have assumed *k*_−2_ = *k*_off_ following Ref. (44). When not explicitly shown, we have used the same parameters as in Ref. (26)*.

*^a^The cross-linking rate *k*_2_ is adapted from Ref. (44) for bivalent receptors*.

**Table 2:** **Estimated mean number of cross-linking events, $N^{\prime }≃N\;e^{2k_{-2^{\tau }}}$, required to elicit a T cell response (SP thymocytes)**.

Ligand	*N*′		
	*τ* (s)	*N* = 10	*N* = 100
4P	1	3	12
(*k*_on_ = 153,691 M^−1^ s^−1^)	4	3	13
(*k*_off_ = 0.0169 s^−1^)	8	3	14
4A	1	7	58
(*k*_on_ = 157,533 M^−1^ s^−1^)	4	~10^3^	~10^4^
(*k*_off_ = 0.8664 s^−1^)	8	~10^6^	~10^7^
4N	1	~10^7^	~10^8^
(*k*_on_ = 149,385 M^−1^ s^−1^)	4	~10^30^	~10^31^
(*k*_off=8.6643_ s^−1^)	8	~10^60^	~10^61^

There is some evidence that, under physiological conditions, the chance of two specific peptides being presented by two MHC molecules in sufficient proximity and long enough to act as a dimer is very small (46). This will make ligand-induced TCR cross-linking a rare event. However, some recent experimental work on the distribution of cognate pMHC molecules on the surface of APCs shows that both for MHC class I (virus infection models), and for MHC class II (antigen uptake via the endocytic route) clusters of cognate pMHC can be detected (19–21).

We also note that ligand concentration is not the only factor that depends on physiological conditions. According to the DRiP hypothesis (28, 29), rapid viral antigen presentation is possible because antigenic peptides originate from defective ribosomal products that have short half-lives. Although this phenomenon affects the time between viral challenge and antigen presentation, we assume it is independent of the subsequent signaling dynamics of T cell activation.

## 3 Results

### 3.1 Distribution of TCR clusters

**Figure 3:**
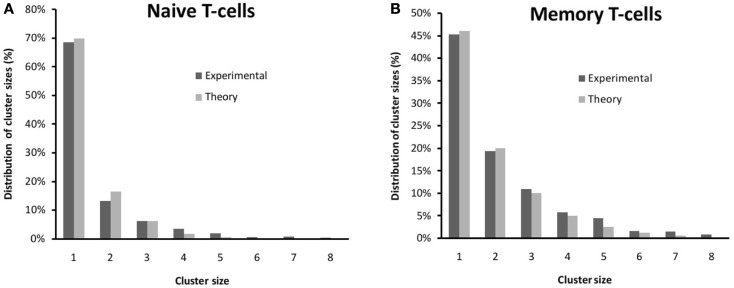
**Comparison between the experimental distribution of clusters (see also Figure 1) and those from Model 1 for (A) naive T cells and (B) memory T cells**. The theoretical distribution has been fitted to equation (2) using a weighted (by the variance) minimum-square regression. The fitted values are *b*_naive_ = 0.32 and *b*_memory_ = 0.55.

The mathematical model described in Section 2.1, or Model 1, allows us to obtain the value of *b* that best fits the experimental data. We have used a weighted (by the variance) minimum-square regression to fit the experimental distributions to equation (2). This kind of fit minimizes the value of *χ*^2^. Thus, in Figure 3, we show the agreement between theory and experiment, with values: *b*_naive_ = 0.32 and *b*_memory_ = 0.55. The difference between *b*_naive_ and *b*_memory_ can be explained by the existence of larger (or at least more localized) lipid rafts on the membrane of memory T cells (50, 51). Thus, the rates *q*_±_could be the effective combination of two mechanisms: one related to the diffusion of receptors on the membrane, and the other related to the aggregation of the receptors at the molecular level. The presence of cholesterol on the membrane changes the diffusion coefficient of the TCR receptors, as receptor diffusion within the raft is inhibited due to protein anchorage (52) and, thus, stabilizes the formation of clusters (a larger value of *b* means that, once two receptors are embedded in the same lipid raft, it is more difficult for them to become separated from each other).

A consequence of Model 1 is that, as the stationary probabilities need to sum up to one, the fraction of clusters of size larger than one is, precisely, *b*. This fraction is 72% higher for memory T cells than for naive T cells: *b*_memory_/*b*_naive_ = 1.72.

### 3.2 Mean time to signal initiation

**Figure 4:**
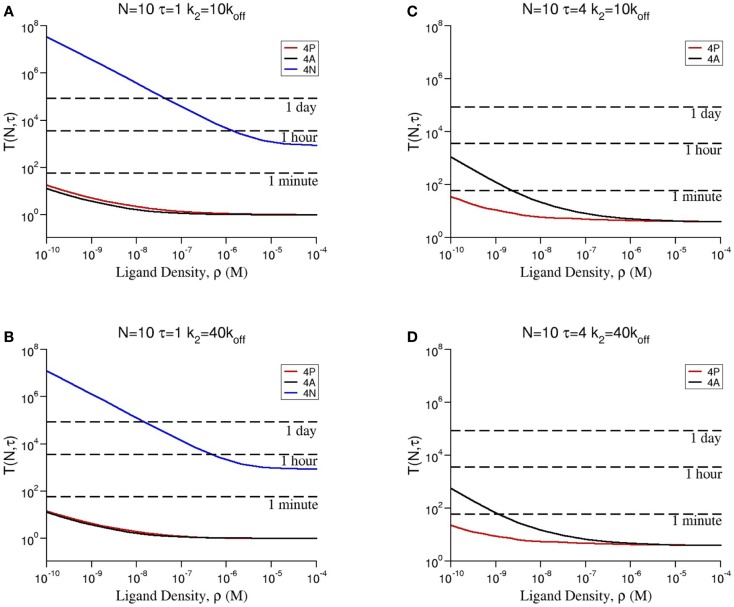
**Dependence of the mean time to signal initiation (MTSI), *T*(*N*, *τ*) to have *N* cross-linked ligand–receptor complexes bound for at least a dwell time *τ* for different model parameters as shown in every panel**. The results have been obtained by making use of a Gillespie algorithm, after averaging over 100 realizations for each set of the parameters, summarized in Table 2 (a python code for the stochastic integration is available upon request). Units of time are seconds. All parameters are taken from Tables 1 and 2 except **(A)** *N* = 10, τ = 1 and *k*_2_ = 10 × *k*_off_; **(B)** *N* = 10, τ = 1 and *k*_2_ = 40 × *k*_off_; **(C)** *N* = 10, τ = 4 and *k*_2_ = 10 × *k*_off_; **(D)** *N* = 10, τ = 4 and *k*_2_ = 40 × *k*_off_.

In Figures 4A–D, we show how the stochastic criterion is able to provide a ligand hierarchy according to their *potency*. Namely, the most agonistic ligand, 4P, elicits a T cell response in times of the order of a few seconds in all cases. On the contrary, the most antagonistic ligand, 4N, takes extremely large times to do so (in practical terms, this means it does not elicit a T cell response). Thus, TCR clustering can enhance the *potency* of ligands, when compared to the monomeric case (26), as experimentally observed and theoretically shown.

**Figure 5:**
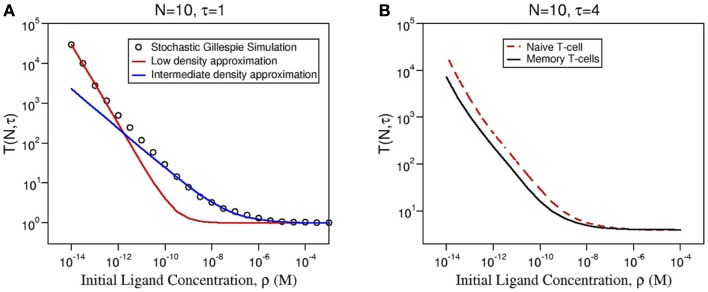
**(A)** Comparison of the numerical solution of Model 2 (Gillespie algorithm with the parameters summarized in Table 2) and the approximate solution [equation (3)] for ligand 4P and the same parameters as in Figure 4. **(B)** Comparison of the mean MTSI for naive (red dashed line) and memory (solid black line) T cells.

Following a similar approach to that of Ref. (26), we can derive an approximate formula for the mean time to signal initiation (MTSI), *T*(*N*, *τ*), for different ranges of ligand concentration, *ρ*. We write (see Figure 5A and Section 5.3 for further details):

(3) $$\begin{array}{l} T\left(N,\tau \right)≃\left\{\begin{array}{ll} \tau \; & \mathrm{at}\;\mathrm{high}\;\mathrm{concentration}\\ \tau +{\left[\frac{N\text{ exp}\left(2k_{-2}\tau \right)}{2\rho N_{B}k_{\mathrm{on}k_{2}}}\right]}^{1∕2}\; & \begin{array}{c} \mathrm{at}\;\mathrm{intermediate}\;\mathrm{concentration} \end{array}\\ \;\\ \tau +\frac{N\exp\left(2k_{-2}\tau \right)}{4\rho N_{B}k_{\mathrm{on}}\left(k_{2}∕k_{-2}\right)}\; & \mathrm{at}\;\mathrm{low}\;\mathrm{concentration}\\ \; \end{array}\right. \end{array}$$

These three regimes correspond to different immunological scenarios. In the case of high concentration of ligand, ligand is in great excess, so that the required number of *signaling units* is reached, almost as soon as the first signaling unit is formed (time of order *τ*). At low ligand concentration, the dynamics is limited by the first binding event, as cross-linking occurs in a slower timescale. So, the MTSI has the same functional form as that for the monomeric case (26). Finally, for intermediate ligand concentration, the competition between binding and cross-linking implies a more complicated mathematical relationship. Of greater relevance to the discussion is the nature of the ligand (with different *k*_on_ and *k*_off_ rates), and the number of TCR clusters on the membrane of the T cell (encoded in the parameter *N_B_* = *b *× *N_R_*, with *N_R_*, the average number of TCRs per T cell, see Table 1).

An expression for the variance of the time to signal initiation (TSI) cannot be provided in a closed form [as done in Ref. (26)]. However, the fact that the variance decreases as the ligand concentration increases, suggests that the mathematical formula for the variance in the monovalent case can provide an upper bound to the present (dimeric) case.

Using equation (3), we also can deduce the role of pre-clustering in the signaling time, or MTSI. As the number of bivalent clusters is *b *× *N_R_*, the larger *b* is, the shorter the response time becomes. The model predicts that, for physiological conditions (not too high ligand concentrations), the ratio of the MTSI for naive and memory T cells is inversely proportional to the ratio of their corresponding values of *b*. Namely, memory cells would respond up to 72% faster than naive ones (Figure 5B).

## 4 Discussion

TCR triggering mechanisms are currently under debate [see, for example, Ref. (53) and (7) for recent reviews]. TCR clustering may be invoked as a description of the experimental results (27). The requirement for multivalent engagement of TCRs by pMHC ligands in CD4^+^ T cells has been widely shown (45, 47, 54, 55). The same requirement was shown in CD8^+^ T cells by Stone and Stern (56).

In this paper, we have made use of the concept of *mean time to signal initiation* (MTSI or stochastic criterion) as a method to quantify the effect of TCR clustering on the timescales of T cell responses and, thus, to compare the behavior of naive and memory T cells. This criterion has also allowed us to compare the results in Section 3 for dimeric binding with those of Ref. (26) for monomeric binding. The introduction of the cross-linked ligand–receptor complex as the minimum *signaling* unit gives the response greater sensitivity to small differences in ligand affinity.

A recent and novel feature of TCR immunology is the existence of TCR nano-clusters that are pre-formed, independently of ligand (3). This suggests that a simple stoichiometric clustering model (oligomerization of free TCRs diffusing on the T cell membrane) is enough to account for the distribution of TCR nano-clusters. In the case of naive T cells, Model 1 predicts an effective non-dimensional parameter, *b* = *q*_+_/*q*_−_, that allows us to explain the experimentally observed TCR cluster distributions. The presence of larger lipid rafts on the membrane of memory T cells might provide support for the different values of *b* for naive and memory cells, *b*_naive_ and *b*_memory_, respectively. It has recently been shown that receptor diffusion within the raft is inhibited due to protein anchorage (52). This reduction in the TCR diffusion coefficient would increase the time required for the receptor to escape from the raft [in a similar fashion as other escape problems (57)]. This escape time is inversely proportional to the diffusion coefficient itself. A smaller TCR diffusivity, as would be the case for memory T cells, will imply a larger residence time in the raft, which in turn will increase the probability of receptor aggregation in a given TCR cluster. A more detailed model of TCR diffusion and aggregation on the T cell membrane will be the subject of future work.

Equation (3) shows the explicit dependence of the MTSI, *T*(*N*, *τ*), on the parameter *N_B_*, for given values of *N* and *τ*. *N_B_* is the average number of dimeric receptor clusters per T cell, so that *N_B_* = *b *× *N_R_*, with *N_R_*, the average number of TCRs per T cell (see Table 1). For large ligand concentration, the predicted T cell response time for memory and naive T cells is the same, and is equal to *τ*. In the case of intermediate concentrations, the MTSI is proportional to $\frac{1}{\sqrt{b}}$. Finally, for low ligand concentration the MTSI is proportional to $\frac{1}{b}$. This implies that, at low ligand concentration, TCR pre-clustering alone can only account for at most 72% of the reduction in the response time between memory and naive T cells. This behavior is illustrated in Figure 5A. This difference is not so large as to be able to account for the observed higher responsiveness of memory T cells. Our results, thus, point to the need for additional mechanisms beyond TCR pre-clustering.

A potential candidate to explain the large differences between memory and naive T cell responses is the conformational change of the CD3 complex (58). This conformational change is essential to enable ITAM phosphorylation and, thus, the transfer of the TCR signal from the ecto-domain to the cytoplasmic tail of the TCR (58). Conformational changes in the CD3 complex occur as a result of the *αβ* hetero-dimer binding to pMHC. These conformational changes allow the subunits of the CD3 complex (the *γϵ* and *δϵ* hetero-dimers and the *ζζ* homo-dimer) to become accessible to Lck, which can then phosphorylate their cytoplasmic domains at the ITAMs, leading to T cell signaling (59). In this way, the ligand-induced conformational change of the receptors can be propagated to all the receptors in the same cluster, so that larger clusters would benefit from this conformational change as a *cascade* [see, for example, Ref. (60) and references therein]. Thus, differences in the distribution of cluster sizes could, indeed, explain the immunological differences between memory and naive cells.

Other membrane receptors also exhibit pre-clustering and ligand-induced receptor cross-linking. For instance, in the case of the vascular endothelial growth factor receptor (VEGFR), it has been shown (61) that there are two distinct pathways to receptor dimerization: (i) dynamic pre-dimerization (as the one described in Model 1), and (ii) ligand-induced receptor dimerization. The main conclusion in Ref. (61) is that both mechanisms are almost indistinguishable at low ligand concentration. However, the first mechanism is more sensitive to changes in the binding affinity at large ligand concentration. Although the biological system studied in Ref. (61) is different from the T cell receptor considered here, their conclusions might be generalized as both receptors are tyrosine kinases.

Bachmann et al. (62, 63) considered a model of diffusion and ligand-induced TCR clustering. Their model suggests that the existence of large enough clusters greatly inhibits subsequent multimer diffusion, thus, reducing the relevance that this mechanism might have. This inhibition might be experimentally tested by exploiting the differences between naive (small and few clusters) and memory (large and many clusters). It will be interesting to make use of the models introduced in this paper to investigate the different roles of ligand binding and cellular activation (62), and TCR turnover (64).

Finally, the existence of TCR pre-clusters [and the knowledge of their membrane distribution given by *π_n_*, equation (2)] can be considered in the kinetic-segregation model (65). In this model, diffusion out of close-contact zones would be inhibited by the existence of nano-clusters, thus, enhancing the number of triggered receptors. In a similar way, consecutive receptor phosphorylation events (66) in TCR nano-clusters would also amplify receptor signaling.

## 5 Materials and Methods

### 5.1 Experiments

Naive CD8^+^ OT-1 T cells, which recognize an ovalbumin-derived peptide presented by the MHC class I molecule H-2K^b^, were isolated from superficial and mesenteric lymph nodes of OT-1 TCR transgenic mice (67), via depletion of CD19^+^ B cells, CD4^+^ helper T cells and CD11b^+^ macrophages, using antibodies and Dynal magnetic beads (Invitrogen). Memory OT-1 T cells were generated by adoptively transferring 10^6^ naive OT-1 T cells into congenic C57BL/6 Ly5.1 Pep3b mice, which were simultaneously immunized with 10^7^ PFU MVA-OVA (68). After 6 months, resting memory OT-1 T cells were isolated from the spleen and lymph nodes of these mice by antibody-mediated depletion of macrophages, B cells, and CD4^+^ T cells, followed by separation of the OT-1 memory T cells from host-derived Ly5.1^+^ CD8^+^ T cells via fluorescence-activated cell sorting, using a Ly5.1-specific antibody. Labeling of cells with the CD3*ϵ*-specific antibody 2C11 and 10 nm gold-conjugated protein-A, replica generation and analysis were performed as previously described (17).

### 5.2 Models of signaling with dimeric and trimeric receptor clusters

In Section 2, we introduced a model in which ligands are bivalent and receptor clusters are dimeric (that is, composed of two monomeric TCRs). This is, of course, a first approximation that neglects the distribution of cluster sizes experimentally observed. Yet, the results of our mathematical study only change in a quantitative way, but not qualitatively, when we include TCR clusters of larger sizes. In this Section, we illustrate this by considering a system in which clusters of size 1, 2, and 3 coexist and the ligands are bivalent. Table 3 provides the notation introduced to describe the molecular species considered in the model, as well as a graphical representation.

**Table 3:** **Summary of variables for a model in which clusters of size 1, 2, and 3 coexist**.

Variable	Description	Molecular representation
*z*_1_	Free monomeric receptor	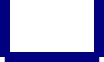
*z*_2_	Free ligand (dimer)	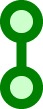
*z*_3_	Ligand-bound to a monomeric receptor	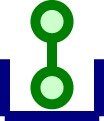
*x*_1_	Free dimeric cluster	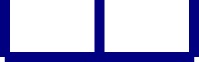
*x*_2_	Same as *z*_2_ (defined for convenience of notation)	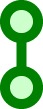
*x*_3_	Ligand singly bound to a dimeric cluster	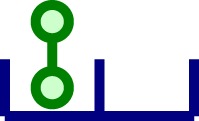
*x*_4_	Two ligands bound to a dimeric cluster	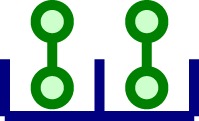
*x*_5_	Cross-linked ligand in a dimeric cluster	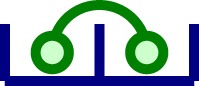
*y*_1_	Free trimeric receptor	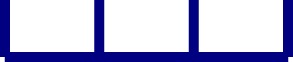
*y*_2_	Same as *z*_2_ (defined for convenience of notation)	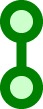
*y*_3_	Ligand singly bound to a trimeric cluster	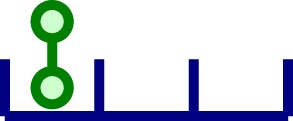
*y*_4_	Two ligands bound to a trimeric cluster	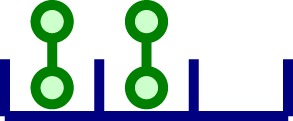
*y*_5_	Cross-linked ligand in a trimeric cluster	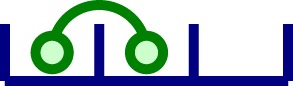
*y*_6_	Three ligands bound to a trimeric cluster	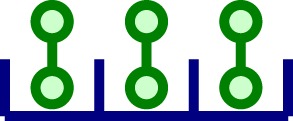
*y*_7_	One ligand singly bound to a trimeric cluster and another cross-linked	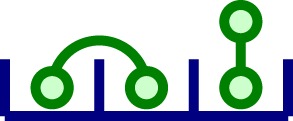

*All the variables correspond to the total number of molecular species (not concentrations). Hence, all the rates in the mathematical model have units of s^−1^*.

At large initial ligand concentration, under the stochastic criterion, the MTSI tends to *τ*. On the other hand, at low initial ligand concentration, the number of receptors, compared to the number of ligands, is so large that we can neglect molecular species *x*_4_, *y*_4_, *y*_6,_ and *y*_7_, which involve more than one bivalent ligand. This has also been confirmed experimentally. Given our stochastic T cell response criterion, in this case, the signaling units correspond to molecular species *x*_5_, *y*_5_, and *y*_7_. Molecular species *z*_1_ and *z*_3_ do not contribute to the T cell response and will be neglected in what follows. Thus, we only need to consider the dynamics of dimeric and trimeric T cell receptor clusters.

We introduce the total number of *signaling units*, $S_{5}\left(t\right)\equiv x_{5}\left(t\right)+y_{5}\left(t\right)$. The set of ordinary differential equations for the model is given by:

$$\begin{array}{llll} & {ẋ}_{1}=-4k_{+}x_{1}x_{2}+k_{\mathrm{off}}\;x_{3},\; & & \;\\ & {ẋ}_{2}=-4k_{+}x_{1}x_{2}+k_{\mathrm{off}}\;x_{3},\; & & \;\\ & {ẋ}_{3}=4k_{+}x_{1}x_{2}-k_{\mathrm{off}}\;x_{3}-k_{2}x_{3}+2k_{-2}x_{5},\; & & \;\\ & {ẋ}_{5}=k_{2}x_{3}-2k_{-2}x_{5},\; & & \;\\ & {ẏ}_{1}=-6k_{+}y_{1}y_{2}+k_{\mathrm{off}}\;y_{3},\; & & \;\\ & {ẏ}_{2}=-6k_{+}y_{1}y_{2}+k_{\mathrm{off}}\;y_{3},\; & & \;\\ & {ẏ}_{3}=6k_{+}y_{1}y_{2}-k_{\mathrm{off}}\;y_{3}-2k_{2}y_{3}+2k_{-2}y_{5},\; & & \;\\ & {ẏ}_{5}=2k_{2}y_{3}-2k_{-2}y_{5},\; & & \; \end{array}$$

where *k*_+_ = *k*_on_/(*VN_A_*), *V* is the volume of the experiment and *N_A_* is Avogadro’s number.

Given the symmetry of the problem, and in the limit of low initial ligand concentration, we will assume that the ratio of *x*_3_ to *y*_3_ is that of the initial ratio of free TCR dimers to free TCR trimers, namely,

(4) $$\frac{y_{3}}{x_{3}}≃\frac{\pi_{3}}{\pi_{2}}=b\;\Rightarrow \;y_{3}≃b\;x_{3}\;,$$

where we have made use of equation (2) to conclude $\frac{\pi_{3}}{\pi_{2}}=b$. Thus, the total number of signaling units, *S*_5_(*t*), obeys the following differential equation

(5) $${Ṡ}_{5}={ẋ}_{5}+{ẏ}_{5}=k_{2}\;\left(1+2b\right)\;x_{3}-2\;k_{-2}\;S_{5}\;.$$

Finally, in the low ligand concentration limit as above, let us introduce $S_{3}\equiv x_{3}+y_{3}$. It is easy to show that equation (5) reduces to

(6) $${Ṡ}_{5}=k_{2}\;\frac{1+2b}{1+b}\;S_{3}-2\;k_{-2}\;S_{5}\;,$$

which is identical to the differential equation for *x*_5_ above, but with *S*_5,3_ replaced by *x*_5,3_, respectively. This means that, except for a pre-factor $\frac{1+2b}{1+b}$ [which, for *b* ∈ (0, 1), is between 1 and 3/2], the study of dimeric and trimeric clusters is reduced to the dimeric case.

### 5.3 A simple formula for the MTSI

The basic idea behind the stochastic criterion is to count the cumulative number of events that may contribute to signaling (26). Here, we calculate the mean number of cross-linking events up to time *t*, *C*(*t*), as the integral,

(7) $$C\left(t\right)=k_{2}\int_{0}^{t}\;x_{3}\left(s\right)\;ds\;.$$

It is possible to obtain an expression for *x*_3_(*t*) with the approximation that the product *x*_1_(*t*)*x*_2_(*t*) is constant, so that the pair of equations for *x*_3_(*t*) and *x*_5_(*t*) can be solved exactly. This yields (69):

(8) $$\begin{array}{llll} C\left(t\right) & =k_{2}\left[\frac{c_{1}}{\lambda_{1}}\left(\lambda_{1}+2k_{-2}\right)\left(e^{\lambda_{1}t}-1\right)\right.\; & & \;\\ & \left.\;+\frac{c_{2}}{\lambda_{2}}\left(\lambda_{2}+2k_{-2}\right)\left(e^{\lambda_{2}t}-1\right)+a_{1}t\right]\;, \end{array}$$

where

$$\begin{array}{llll} c_{1} & =\frac{-4\lambda_{2}k_{\mathrm{on}}\rho N_{B}}{\left(\lambda_{2}-\lambda_{1}\right)\left(4k_{\mathrm{on}}\rho k_{2}+2k_{\mathrm{off}}k_{-2}+8k_{\mathrm{on}}\rho k_{-2}\right)}\;,\; & & \;\\ c_{2} & =\frac{4\lambda_{1}k_{\mathrm{on}}\rho N_{B}}{\left(\lambda_{2}-\lambda_{1}\right)\left(4k_{\mathrm{on}}\rho k_{2}+2k_{\mathrm{off}}k_{-2}+8k_{\mathrm{on}}\rho k_{-2}\right)}\;,\; & & \;\\ \lambda_{1,2} & =\frac{1}{2}\left(-4k_{\mathrm{on}}\rho -k_{\mathrm{off}}-k_{2}-2k_{-2}\right.\; & & \;\\ & \left.\;\pm {\left[{\left(4k_{\mathrm{on}}\rho +k_{\mathrm{off}}-k_{2}-2k_{-2}\right)}^{2}+4k_{\mathrm{off}}k_{2}\right]}^{1∕2}\right)\;,\; & & \;\\ a_{1} & =\frac{8k_{-2}k_{\mathrm{on}}\rho N_{B}}{4k_{\mathrm{on}}\rho k_{2}+2k_{\mathrm{off}}k_{-2}+8k_{\mathrm{on}}\rho k_{-2}}\;,\; & & \;\\ a_{2} & =\frac{4k_{2}k_{\mathrm{on}}\rho N_{B}}{4k_{\mathrm{on}}\rho k_{2}+2k_{\mathrm{off}}k_{-2}+8k_{\mathrm{on}}\rho k_{-2}}\;,\; & & \; \end{array}$$

and *N_B_* is the number of dimeric receptors. The MTSI is then given by the solution of the equation *C*(*T*(*N*, *τ*)–*τ*) = *N* exp(2*k*_−2_*τ*).

The expressions in equation (3) are obtained from equation (8) in the appropriate regimes. At low ligand concentration, *C*(*t*) is simply proportional to time: $C\left(t\right)≃k_{2}a_{1}t$, so that *C*(*T *− *τ*) = *k*_2_*a*_1_(*T *− *τ*) = *N* exp(2*k*_−2_*τ*). When *λ*_1,2_*τ* ≪ 1, on the other hand, the first non-zero term in a Taylor expansion of *C*(*t*) in time is quadratic: *C*(*t*) *∝ t*^2^. This provides the exponent 1/2 in the second line of equation (3).

## Conflict of Interest Statement

The authors declare that the research was conducted in the absence of any commercial or financial relationships that could be construed as a potential conflict of interest.

## References

[1] Huppa JB Davis MM T-cell-antigen recognition and the immunological synapse. Nat Rev Immunol (2003) 3(12):973–83 10.1038/nri1245 14647479

[2] Batista FD Dustin ML Cell: cell interactions in the immune system. Immunol Rev (2013) 251(1):7–12 10.1111/imr.12025 23278736

[3] Schamel WW Alarcón B Organization of the resting TCR in nanoscale oligomers. Immunol Rev (2013) 251(1):13–20 10.1111/imr.12019 23278737

[4] Allard JF Dushek O Coombs D van der Merwe PA Mechanical modulation of receptor-ligand interactions at cell-cell interfaces. Biophys J (2012) 102(6):1265–73 10.1016/j.bpj.2012.02.006 22455909 PMC3309404

[5] Lillemeier BF Mörtelmaier MA Forstner MB Huppa JB Groves JT Davis MM TCR and Lat are expressed on separate protein islands on T cell membranes and concatenate during activation. Nat Immunol (2009) 11(1):90–6 10.1038/ni.1832 20010844 PMC3273422

[6] Edwards LJ Zarnitsyna VI Hood JD Evavold BD Zhu C Insights into t cell recognition of antigen: significance of two-dimensional kinetic parameters. Front Immunol (2012) 3:86 10.3389/fimmu.2012.00086 22566966 PMC3342060

[7] Zarnitsyna V Zhu C T cell triggering: insights from 2D kinetics analysis of molecular interactions. Phys Biol (2012) 9(4):045005 10.1088/1478-3975/9/4/045005 22871794

[8] McKeithan T Kinetic proofreading in T-cell receptor signal transduction. Proc Natl Acad Sci U S A (1995) 92(11):5042 10.1073/pnas.92.11.5042 7761445 PMC41844

[9] Kalergis AM Boucheron N Doucey M-A Palmieri E Goyarts EC Vegh Z Efficient T cell activation requires an optimal dwell-time of interaction between the TCR and the pMHC complex. Nat Immunol (2001) 2(3):229–34 10.1038/85286 11224522

[10] Matis LA Glimcher LH Paul WE Schwartz RH Magnitude of response of histocompatibility-restricted T-cell clones is a function of the product of the concentrations of antigen and IA molecules. Proc Natl Acad Sci U S A (1983) 80(19):6019–23 10.1073/pnas.80.19.6019 6310611 PMC534351

[11] Valitutti S Müller S Cella M Padovan E Lanzavecchia A Serial triggering of many T-cell receptors by a few peptide MHC complexes. Nature (1995) 375(6527):148–51 10.1038/375148a0 7753171

[12] Friedl P Gunzer M Interaction of T cells with APCS: the serial encounter model. Trends Immunol (2001) 22(4):187–91 10.1016/S1471-4906(01)01869-5 11274922

[13] Dushek O Das R Coombs D A role for rebinding in rapid and reliable T cell responses to antigen. PLoS Comput Biol (2009) 5(11):e1000578 10.1371/journal.pcbi.1000578 19956745 PMC2775163

[14] Daniels MA Teixeiro E Gill J Hausmann B Roubaty D Holmberg K Thymic selection threshold defined by compartmentalization of RAS/MAPK signalling. Nature (2006) 444(7120):724–9 10.1038/nature05269 17086201

[15] Valitutti S Coombs D Dupré L The space and time frames of T cell activation at the immunological synapse. FEBS Lett (2010) 584(24):4851–7 10.1016/j.febslet.2010.10.010 20940018

[16] Smith-Garvin JE Koretzky GA Jordan MS T cell activation. Annu Rev Immunol (2009) 27:591 10.1146/annurev.immunol.021908.132706 19132916 PMC2740335

[17] Kumar R Ferez M Swamy M Arechaga I Rejas MT Valpuesta JM Increased sensitivity of antigen-experienced T cells through the enrichment of oligomeric T cell receptor complexes. Immunity (2011) 35(3):375–87 10.1016/j.immuni.2011.08.010 21903423

[18] Schamel WW Arechaga I Risueño RM van Santen HM Cabezas P Risco C Coexistence of multivalent and monovalent TCRs explains high sensitivity and wide range of response. J Exp Med (2005) 202(4):493–503 10.1084/jem.20042155 16087711 PMC2212847

[19] Lu X Gibbs JS Hickman HD David A Dolan BP Jin Y Endogenous viral antigen processing generates peptide-specific MHC class I cell-surface clusters. Proc Natl Acad Sci U S A (2012) 109(38):15407–12 10.1073/pnas.1208696109 22949678 PMC3458372

[20] Ferez M Castro M Alarcon B van Santen HM Cognate peptide-MHC complexes are expressed as tightly apposed nanoclusters in virus-infected cells to allow tcr crosslinking. J Immunol (2014) 192(1):52–8 10.4049/jimmunol.1301224 24307729

[21] Bosch B Heipertz EL Drake JR Roche PA Major histocompatibility complex (MHC) class II-peptide complexes arrive at the plasma membrane in cholesterol-rich microclusters. J Biol Chem (2013) 288(19):13236–42 10.1074/jbc.M112.442640 23532855 PMC3650363

[22] Goldstein B Faeder JR Hlavacek WS Mathematical and computational models of immune-receptor signalling. Nat Rev Immunol (2004) 4(6):445–56 10.1038/nri1374 15173833

[23] Alarcón B Swamy M van Santen HM Schamel WW T-cell antigen-receptor stoichiometry: pre-clustering for sensitivity. EMBO Rep (2006) 7(5):490–5 10.1038/sj.embor.7400682 16670682 PMC1479560

[24] Bray D Levin MD Morton-Firth CJ Receptor clustering as a cellular mechanism to control sensitivity. Nature (1998) 393(6680):85–8 10.1038/30018 9590695

[25] Slifka MK Whitton JL Functional avidity maturation of CD8^+^ T cells without selection of higher affinity TCR. Nat Immunol (2001) 2(8):711–7 10.1038/90650 11477407

[26] Currie J Castro M Lythe G Palmer E Molina-París C A stochastic T cell response criterion. J R Soc Interface (2012) 9(76):2856–70 10.1098/rsif.2012.0205 22745227 PMC3479899

[27] van der Merwe P Dushek O Mechanisms for T cell receptor triggering. Nat Rev Immunol (2010) 11(1):47–55 10.1038/nri2887 21127503

[28] Yewdell JW Drips solidify: progress in understanding endogenous MHC class I antigen processing. Trends Immunol (2011) 32(11):548–58 10.1016/j.it.2011.08.001 21962745 PMC3200450

[29] Rock KL Farfán-Arribas DJ Colbert JD Goldberg AL Re-examining class-I presentation and the DRiP hypothesis. Trends Immunol (2014). 10.1016/j.it.2014.01.002 24566257 PMC3986829

[30] Antón LC Yewdell JW Translating DRiPs: MHC class I immunosurveillance of pathogens and tumors. J Leukoc Biol (2014). 10.1189/jlb.1113599 24532645 PMC3958739

[31] Stone J Cochran J Stern L T-cell activation by soluble MHC oligomers can be described by a two-parameter binding model. Biophys J (2001) 81(5):2547–57 10.1016/S0006-3495(01)75899-7 11606269 PMC1301723

[32] Stone J Chervin A Kranz D T-cell receptor binding affinities and kinetics: impact on T-cell activity and specificity. Immunology (2009) 126(2):165–76 10.1111/j.1365-2567.2008.03015.x 19125887 PMC2632691

[33] Coombs D Dushek O Merwe P A review of mathematical models for T cell receptor triggering and antigen discrimination. In: Molina-París C Lythe G, editors. Mathematical Models and Immune Cell Biology. New York: Springer (2011). p. 25–45

[34] Coombs D Kalergis AM Nathenson SG Wofsy C Goldstein B Activated TCRs remain marked for internalization after dissociation from pMHC. Nat Immunol (2002) 3(10):926–31 10.1038/ni838 12244312

[35] Choudhuri K Dustin ML Signaling microdomains in T cells. FEBS Lett (2010) 584(24):4823–31 10.1016/j.febslet.2010.10.015 20965175 PMC3870024

[36] Yokosuka T Saito T The immunological synapse, TCR microclusters, and T cell activation. In: Saito T Batista FD, editors. Immunological Synapse. Berlin Heidelberg: Springer (2010). p. 81–107 10.1007/978-3-642-03858-7_5 19960310

[37] Torres J Briggs JA Arkin IT Multiple site-specific infrared dichroism of CD3-*ζ*, a transmembrane helix bundle. J Mol Biol (2002) 316(2):365–74 10.1006/jmbi.2001.5267 11851344

[38] Kuhns MS Girvin AT Klein LO Chen R Jensen KD Newell EW Evidence for a functional sidedness to the *αβ*TCR. Proc Natl Acad Sci U S A (2010) 107(11):5094–9 10.1073/pnas.1000925107 20202921 PMC2841884

[39] Norris JR Markov Chains. Cambridge University Press (1998).

[40] Taylor H Karlin S An Introduction to Stochastic Modeling. San Diego: Academic Press (1998).

[41] Janeway CA Jr Ligands for the T-cell receptor: hard times for avidity models. Immunol Today (1995) 16(5):223–5 10.1016/0167-5699(95)80163-4 7779252

[42] Ma Z Sharp KA Janmey PA Finkel TH Surface-anchored monomeric agonist pMHCs alone trigger TCR with high sensitivity. PLoS Biol (2008) 6(2):e43 10.1371/journal.pbio.0060043 18303949 PMC2253636

[43] Huang J Brameshuber M Zeng X Xie J Li QJ Chien YH A single peptide-major histocompatibility complex ligand triggers digital cytokine secretion in CD4+ T cells. Immunity (2013) 39:846–57 10.1016/j.immuni.2013.08.036 24120362 PMC3846396

[44] Stone J Artyomov M Chervin A Chakraborty A Eisen H Kranz D Interaction of streptavidin-based peptide-MHC oligomers (tetramers) with cell-surface TCRs. J Immunol (2011) 187(12):6281–90 10.4049/jimmunol.1101734 22102724 PMC3237744

[45] Abastado J-P Lone Y-C Casrouge A Boulot G Kourilsky P Dimerization of soluble major histocompatibility complex-peptide complexes is sufficient for activation of T cell hybridoma and induction of unresponsiveness. J Exp Med (1995) 182(2):439–47 10.1084/jem.182.2.439 7629504 PMC2192121

[46] Cochran JR Cameron TO Stern LJ The relationship of MHC-peptide binding and T cell activation probed using chemically defined MHC class II oligomers. Immunity (2000) 12(3):241–50 10.1016/S1074-7613(00)80177-6 10755611

[47] Boniface JJ Rabinowitz JD Wülfing C Hampl J Reich Z Altman JD Initiation of signal transduction through the T cell receptor requires the multivalent engagement of peptide/MHC ligands. Immunity (1998) 9(4):459–66 10.1016/S1074-7613(00)80629-9 9806632

[48] Palmer E Naeher D Affinity threshold for thymic selection through a T-cell receptor-co-receptor zipper. Nat Rev Immunol (2009) 9(3):207–13 10.1038/nri2469 19151748

[49] Gillespie D Exact stochastic simulation of coupled chemical reactions. J Phys Chem (1977) 81:2340–61 10.1021/j100540a008

[50] Brumeanu T-D Preda-Pais A Stoica C Bona C Casares S Differential partitioning and trafficking of GM gangliosides and cholesterol-rich lipid rafts in thymic and splenic CD4 T cells. Mol Immunol (2007) 44(4):530–40 10.1016/j.molimm.2006.02.008 16597465

[51] Kersh EN Kaech SM Onami TM Moran M Wherry EJ Miceli MC TCR signal transduction in antigen-specific memory CD8 T cells. J Immunol (2003) 170(11):5455–63 12759421 10.4049/jimmunol.170.11.5455

[52] Lingwood D Simons K Lipid rafts as a membrane-organizing principle. Science (2010) 327(5961):46–50 10.1126/science.1174621 20044567

[53] Robert P Aleksic M Dushek O Cerundolo V Bongrand P van der Merwe P Kinetics and mechanics of two-dimensional interactions between T cell receptors and different activating ligands. Biophys J (2012) 102(2):248–57 10.1016/j.bpj.2011.11.4018 22339861 PMC3260781

[54] Cochran JR Stern LJ A diverse set of oligomeric class II MHC-peptide complexes for probing T-cell receptor interactions. Chem Biol (2000) 7(9):683–96 10.1016/S1074-5521(00)00019-3 10980449

[55] Dushek O Goyette J Merwe PA Non-catalytic tyrosine-phosphorylated receptors. Immunol Rev (2012) 250(1):258–76 10.1111/imr.12008 23046135

[56] Stone JD Stern LJ CD8 T cells, like CD4 T cells, are triggered by multivalent engagement of TCRs by MHC-peptide ligands but not by monovalent engagement. J Immunol (2006) 176(3):1498–505 16424178 10.4049/jimmunol.176.3.1498

[57] Day M Lythe G Timescales of the adaptive immune response. In: Molina-París C Lythe G, editors. Mathematical Models and Immune Cell Biology. New York: Springer (2011). p. 351–61

[58] Minguet S Swamy M Alarcón B Luescher IF Schamel WW Full activation of the T cell receptor requires both clustering and conformational changes at CD3. Immunity (2007) 26(1):43–54 10.1016/j.immuni.2006.10.019 17188005

[59] Marks F Klingmüller U Müller-Decker K Cellular Signal Processing: An Introduction to the Molecular Mechanisms of Signal Transduction. New York, NY: Garland Science (2009).

[60] Blanco R Alarcón B TCR nanoclusters as the framework for transmission of conformational changes and cooperativity. Front Immunol (2012) 3:115 10.3389/fimmu.2012.00115 22582078 PMC3348506

[61] Mac Gabhann F Popel AS Dimerization of VEGF receptors and implications for signal transduction: a computational study. Biophys Chem (2007) 128(2–3):125–39 10.1016/j.bpc.2007.03.010 17442480 PMC2711879

[62] Bachmann MF Salzmann M Oxenius A Ohashi PS Formation of TCR dimers/trimers as a crucial step for T cell activation. Eur J Immunol (1998) 28(8):2571–9 10.1002/(SICI)1521-4141(199808)28:08<2571::AID-IMMU2571>3.0.CO;2-T 9710234

[63] Bachmann MF Ohashi PS The role of T-cell receptor dimerization in T-cell activation. Immunol Today (1999) 20(12):568–76 10.1016/S0167-5699(99)01543-1 10562708

[64] Sousa J Carneiro J A mathematical analysis of tcr serial triggering and down-regulation. Eur J Immunol (2000) 30:3219–27 10.1002/1521-4141(200011)30:11<3219::AID-IMMU3219>3.0.CO;2-7 11093137

[65] Davis SJ van der Merwe PA The kinetic-segregation model: TCR triggering and beyond. Nat Immunol (2006) 7(8):803–9 10.1038/ni1369 16855606

[66] Mukhopadhyay H Cordoba S-P Maini PK van der Merwe PA Dushek O Systems model of T cell receptor proximal signaling reveals emergent ultra- sensitivity. PLoS Comput Biol (2013) 9(3):e1003004 10.1371/journal.pcbi.1003004 23555234 PMC3610635

[67] Hogquist KA Jameson SC Heath WR Howard JL Bevan MJ Carbone FR T cell receptor antagonist peptides induce positive selection. Cell (1994) 76(1):17–27 10.1016/0092-8674(94)90169-4 8287475

[68] El-Gogo S Staib C Meyr M Erfle V Sutter G Adler H Recombinant murine gammaherpesvirus 68 (MHV-68) as challenge virus to test efficacy of vaccination against chronic virus infections in the mouse model. Vaccine (2007) 25(20):3934–45 10.1016/j.vaccine.2007.02.054 17433507

[69] Currie J Stochastic Modelling of TCR Binding. Ph.D. thesis, Leeds: University of Leeds (2012).

